# A Quality Improvement Project to Improve Periprosthetic Fracture Management at District General Hospitals

**DOI:** 10.7759/cureus.31937

**Published:** 2022-11-27

**Authors:** Ashwin Bhadresha, Chiranjit De, Hassan El Tagy, Venkata Neelapala, Manoj Veettil

**Affiliations:** 1 Trauma and Orthopaedics, The Royal London Hospital, London, GBR; 2 Trauma and Orthopaedics, William Harvey Hospital, Ashford, GBR; 3 Trauma and Orthopaedics, Sandwell General Hospital, Birmingham, GBR

**Keywords:** hip fracture, quality improvement, district general hospital, management, periprosthetic fractures

## Abstract

Introduction

The incidence of periprosthetic femoral fractures (PPF) is expected to rise by 4.6% every decade over the next 30 years. The risk of mortality for patients who sustained a PPF was found to be similar to the mortality rate after a native hip fracture, and so The National Institute for Health and Care Excellence (NICE) guidelines advocate the timely management and mobilisation for patients with PPFs. Patient outcomes following these complex surgeries can be highly variable owing to the variability in regional practice and service delivery.

This study aimed to review the management trend and outcomes of periprosthetic fractures (PPFs) involving hip and knee prostheses at a busy district general hospital in order to improve the overall efficacy in managing these complex fractures.

Methods

This retrospective study included 67 patients who presented to a single district general hospital during a two-year period. Data was collected on demographic profile, further onward referral to a tertiary centre, management (operative versus conservative), timing of surgery, complications, length of stay, implant survivorship, 30-day, one-year, and two-year mortality rate.

Results

Out of the total of 67 PPFs, 51 (76%) were managed operatively, and 16 (24%) were managed conservatively. Of the operatively managed PPFs, 49 (96%) were managed locally at the district general hospital, and two (4%) were managed at the tertiary centre. Eighteen patients (37%) underwent both revision and fixation, whilst 31 (63%) underwent fixation alone. The mortality rates at 30 days, one year, and two years were 10.4%, 20.9%, and 25.4%, respectively. For PPF patients managed operatively, the mean time taken from presentation to operation was 89.2 hours. The overall mean length of hospital stay for all patients was 23.6 days. Eight patients suffered complications. The implant survivorship at two years was 98%.

Conclusion

This study adds objective support for the successful operative management of PPFs at district general hospitals. However, improvement is required in service delivery and the efficacy of management. This could be achieved by a national database for PPFs, improved resource allocation, and prompt logistical support.

## Introduction

Joint arthroplasty has revolutionised the treatment of severely arthritic joints since the advent of total hip replacement. With an ageing population, more arthroplasty operations are being performed annually, resulting in an increased prevalence of periprosthetic fractures (PPFs) [1]. Projection models estimate that the incidence of PPFs is expected to rise by 4.6% every decade over the next 30 years, and according to the UK’s National Joint Registry, over 12,000 hip revision surgeries have been performed since 2003 due to hip PPFs [2,3]. The actual number of PPFs, however, exceeds that because the registry only records cases that undergo revision arthroplasty. It does not include fixation or cases managed conservatively [1]. It is, therefore, difficult to comprehensively assess the increasing technical, clinical, and economic burden of PPFs on our healthcare system. Periprosthetic fractures are complex injuries, and there is still uncertainty over the indications for fixation or revision. The various decisive factors on the choice of management involve multiple patient-dependent and independent factors. The Vancouver classification system is most widely utilised to describe these fractures and guide treatment [4,5].

The risk of mortality for patients who sustained a PPF was found to be similar to the mortality rate after a native hip fracture. The National Institute for Health and Care Excellence (NICE) guidelines advocate the timely management and mobilisation of patients with PPFs, similar to that of hip fractures [6-8]. Patient outcomes following these complex surgeries can be highly variable owing to the variability in regional practice and service delivery.

On conducting a literature review, few district general hospitals have published their experience of managing PPFs. The aim of this study, therefore, was to review the management trend and outcomes of PPFs involving hip and knee prostheses at a busy district general hospital in order to improve the overall efficacy in managing these complex fractures.

## Materials and methods

This single-centre retrospective observational study included 67 patients (20 male, 47 female) who presented to Sandwell General Hospital during a two-year follow-up period from August 2018 to August 2020. Data was extracted from the digital medical record system in line with local clinical governance protocols, and we obtained IRB approval. The data parameters comprised of a demographic profile, further onward referral to a tertiary centre (The Royal Orthopaedic Hospital, Birmingham), management (operative versus conservative), timing of surgery, complications, length of stay, implant survivorship, 30-day, one-year, and two-year mortality rate. Patient management was broadly based on the Vancouver and Unified Classification System, which incorporates the site of the fracture, stability of prosthesis, and bone quality [4,5,9]. Inclusion criteria consisted of femoral fractures that occurred around a primary or revision total hip arthroplasty, hip hemiarthroplasty, and primary or revision total knee arthroplasty. Exclusion criteria consisted of PPFs of the upper limb and those involving intramedullary nails. 

Primary outcome measures included management trend, mortality (30-day, one-year, and two-year), and time to surgery. Secondary outcome measures included complications and length of hospital stay.

## Results

The demographic profile of the study population is summarized in Table 1. During the study, a total of 67 patients (20 male, 47 female) were included. The study group consisted of 36 patients with PPFs of total hip arthroplasty and 31 patients presenting with PPFs involving total knee arthroplasty.

**Table 1:** Demographic profile of the study population

Total patients (N)	67
Mean age (years)	82.5
Gender distribution	
Male	20 (29.9%)
Female	47 (70.1%)
Type of periprosthetic fracture	
Total hip arthroplasty	36
Total knee arthroplasty	31

Primary outcome measures

**Figure 1:**
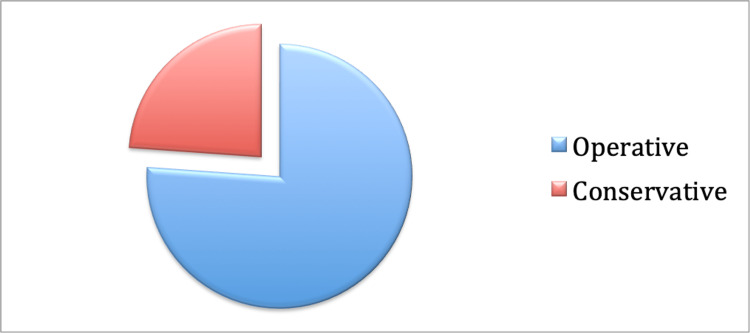
Pie chart showing the breakdown of operative versus conservative management of periprosthetic fractures

**Figure 2:**
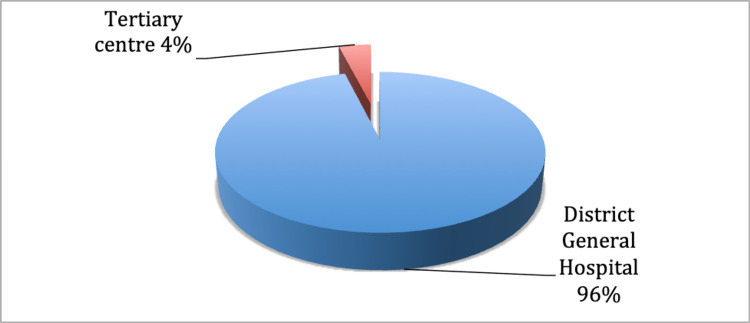
Pie chart showing the breakdown of operatively managed periprosthetic fractures

The management trend of the PPFs is demonstrated in Figure 1. Out of the total of 67 PPFs, 51 (76%) were managed operatively, and 16 (24%) were managed conservatively. A further breakdown of the operatively managed PPFs is shown in Figure 2. This shows that out of the 51 PPFs managed operatively, 49 (96%) were managed locally at the district general hospital. Only two (4%) were managed at the tertiary centre. Out of the 49 PPFs operated at Sandwell General Hospital, 18 patients (37%) underwent both revision and fixation, whilst 31 (63%) underwent fixation alone.

The mortality rates at 30 days, one year, and two years were 10.4%, 20.9%, and 25.4%, respectively. For PPF patients managed operatively, the mean time taken from presentation to a clinical care decision was 32.1 hours. The mean time taken from presentation to operation was 89.2 hours. For patients managed conservatively, the mean time taken from presentation to implementation of the final plan was 88.3 hours.

Secondary outcome measures

**Figure 3:**
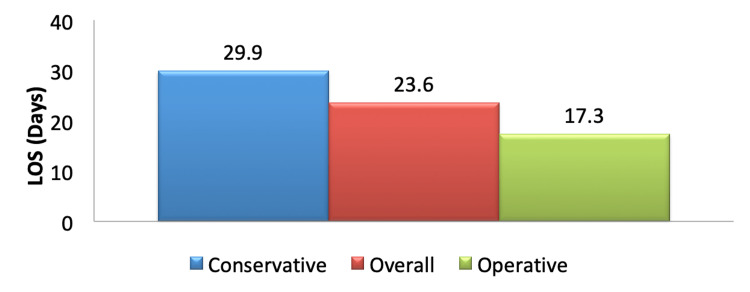
Mean length of hospital stay LOS - length of stay

The overall mean length of hospital stay for all patients was 23.6 days. The mean length of hospital stay for patients who had an operation was 17.3 days. The mean length of hospital stay for patients managed conservatively was 29.9 days. This is summarized in Figure 3.

Table 2 summarises the complications with the operated PPFs. Of the three cases related to infection, two cases were due to a superficial wound infection. One case was due to a deep wound infection. All three cases underwent debridement and wound washout, and the implant was retained in all cases. Two patients suffered post-operative dislocations; one required a manipulation under anaesthetic, and the other required an open reduction and an adductor tenotomy. There were no further prosthesis-related complications during subsequent follow-ups, and the implant was retained in both cases. One patient who underwent a total knee arthroplasty needed implant revision due to aseptic loosening of the femoral component. The implant survivorship at two years was, therefore, 98%.

**Table 2:** Summary of complications

Complication	N
Infection	3 (6.1%)
Dislocation	2 (4%)
Further fractures	2 (4%)
Implant failure	1 (2%)

## Discussion

This study provides valuable insight into the management trend of PPFs at a busy district general hospital. The key findings of this study are that 96% of the operative intervention for PPFs was performed at the district general hospital. There was also a low complication rate, with complications also being managed locally. We found that there was a significant delay to surgery, with only 22.5% of patients being operated on within 36 hours of presentation. Moreover, we found that the mortality rate is comparable to the fractured neck of femur patients at 30 days, but at the 1-year and 2-year follow-ups, the mortality rate is lower compared to hip fractures.

The number of patients admitted to hospitals in England with PPFs is approximately 450 per month, and there is a significant burden on the healthcare system due to the prolonged length of stay and increased dependency on discharge, with 25% unable to return directly to their normal residence [10]. This has caused an increased financial burden, with the mean cost of treatment per patient being £23,649, with the ward cost being responsible for about 80% of the total cost. This highlights that it is the rehabilitation period, the complication rates, and the total length of stay that mostly affect the overall cost [11]. It is, therefore, crucial to manage these fractures efficiently. Non-operative treatment of PPFs has been associated with poor outcomes (malunions, non-unions, medical complications) except in cases where the patient is too ill to undergo any surgical intervention. With the further advances in implants and revision surgery, operative intervention is nowadays the choice of treatment [12].

Mudiganty et al. noted a slightly lower rate of 75% of PPFs being managed locally [13]. They reviewed the hub and spoke model of PPFs, which is designed to ensure that patients are reviewed by the right specialist in an appropriate time frame. They noted that close communication between the hub and spoke hospitals is crucial to reduce inappropriate patient transfers and to ensure a timely transfer [13]. Although this model may allow for a more multi-disciplinary approach with input from the hub units, it must be taken into account that there are significant delays in definitive operative treatment with patients accepted for transfer to their respective hub unit, which will result in an increased morbidity and mortality rate. With the vast majority of patients being managed locally at our district general hospital (96%), it was also interesting to note that complications relating to PPFs were also managed in house instead of being transferred to the hub unit. This demonstrates the preserve of surgeon skill mix in the spoke units.

We found that only 22.5% of patients are being operated on within 36 hours. The NICE guidelines recommend that PPFs should be treated promptly like hip fractures; thus, this significant delay to surgery, with the mean time to operation being 89.2 hours, falls significantly short from the NICE guidelines [8]. A delay in hip fracture surgery over 24 hours is associated with increased mortality, morbidity, and length of stay [14]. Whilst the PPFs are complex cases necessitating thorough planning, delays should be minimised. Baggott et al. found longer surgical waiting times in patients who underwent proximal PPF revision rather than proximal PPF fixation [15]. Part of the surgical delay may be attributable to the fact that successful management of these fractures requires a multi-faceted approach with pre-operative planning, revision templating, and verification of the availability of the required surgical kits. The overall aim of the procedure is to be able to restore anatomical alignment with a stable implant, maintain bone stock, and to allow early patient mobilisation [12].

The mortality rates at 30 days, one year, and two years were 10.4%, 20.9%, and 25.4%, respectively. This is in line with the one-year mortality rates in the current literature, reported as being between 11% and 20% [16,17]. The 30-day mortality rate was similar to that of native hip fractures (8.3%), according to the National Hip Fracture Database [18]. The one-year mortality rate for native hip fractures was lower at 15.9% [7]. Our study also demonstrated an implant survivorship of 98% at two years, which is in keeping with the current literature.

One limitation of this study is that we did not include standardised data on patient-reported outcome measures (PROMS). However, PROMS are not routinely collected for patients with PPFs at our institution. This study could be improved by collecting and including data from other district general hospitals to provide a larger sample size. However, with our two-year follow period, we deemed our sample size of 67 patients to be sufficient for the scope of this study. Moreover, the primary aim of this study was to provide evidence on the management trend for PPFs at a district general hospital.

## Conclusions

In conclusion, we found that 96% of PPFs were managed locally at our district general hospital. There was a low complication rate, and all complications were also managed locally. This study, therefore, adds objective support for the successful operative management of PPFs at district general hospitals. However, it is clear that we need to improve the delivery and efficacy of management due to the significant delay in surgery and prolonged length of hospital stay. A database specifically for PPFs, similar to the NHFD for the neck of femur fractures, would confer superior operational efficacy, as would improved resource allocation and prompt logistical support.
